# NLR and BMI are independent predictors of postoperative drainage volume in macromastia patients following reduction mammoplasty

**DOI:** 10.3389/fsurg.2025.1667856

**Published:** 2025-10-10

**Authors:** Chong Wu, Lin Zhao, Jian Wang, Hongwei Liang, Ye Tao

**Affiliations:** The Fifth Clinical Medical College of Henan University of Chinese Medicine (Zhengzhou People’s Hospital), Zhengzhou, Henan, China

**Keywords:** reduction mammaplasty, postoperative drainage, neutrophil-to-lymphocyte ratio (NLR), obesity, precision monitoring

## Abstract

**Background:**

Accurate prediction of post-reduction mammoplasty drainage volume is critical for optimizing postoperative care and reducing complication risks in patients with macromastia. This study aimed to identify key predictors of total postoperative drainage volume. We further investigated whether these predictors demonstrate consistent effects across diverse populations or subgroups, thereby providing evidence to support personalized management of postoperative drainage.

**Methods:**

Clinical data from 69 macromastia patients were analyzed, including preoperative and postoperative variables such as body mass index (BMI), neutrophil-to-lymphocyte ratio (NLR), lymphocyte-to-monocyte ratio (LMR), and postoperative differential blood cell counts (e.g., postoperative neutrophils, lymphocytes, and monocytes). Data were summarized using descriptive statistics. Variables significantly associated with total drainage volume were screened via Spearman's correlation analysis. Univariate and multivariate regression analyses were subsequently performed to identify independent predictors. Additionally, stratified subgroup analyses based on BMI and age were conducted to assess the consistency of predictor effects.

**Results:**

Univariate and correlation analyses revealed significant positive associations between total drainage volume and both BMI (Spearman's *ρ* = 0.564, *P* < 0.0001) and postoperative NLR (Spearman's *ρ* = 0.506, *P* < 0.0001). Multivariate regression confirmed BMI (*P* < 0.001) and postoperative NLR (*P* = 0.033) as independent and significant predictors of postoperative drainage volume. Furthermore, stratified analyses demonstrated consistent predictive effects for BMI and postoperative NLR across all BMI and age subgroups (*P* < 0.05), with no significant heterogeneity observed.

**Conclusion:**

This study identifies BMI and postoperative NLR as independent predictors of total postoperative drainage volume, highlighting their clinical utility. The consistent predictive performance of these factors across BMI and age subgroups supports their broad applicability. These findings provide evidence-based support for personalized drainage management strategies and offer critical insights for clinical practice.

## Introduction

Macromastia is a common breast developmental disorder characterized by excessive breast enlargement that causes physical and psychological symptoms (e.g., dorsal/neck pain, skin irritation). The diagnostic criteria for macromastia in this study were based on clinical manifestations (symptoms caused by breast weight) and imaging evaluation (breast volume exceeding 800 mL or glandular tissue resection weight ≥200 g per side). This condition frequently causes physical and psychological discomfort, including dorsal/neck pain, skin irritation, and psychosocial distress. While its etiology remains incompletely elucidated, genetic factors, endocrine dysregulation, and obesity are implicated contributors (1–4).

Surgical intervention is the only definitive treatment to alleviate symptoms and significantly improve both physical complaints (e.g., shoulder/back pain, headaches) and psychosocial well-being (5–7). Reduction mammoplasty involves resection of excess glandular tissue, fat, and skin to achieve proportionate breast size and contour. Common techniques include: Free nipple-areola complex (NAC) grafting for severe ptosis or macromastia, though it may compromise aesthetic outcomes (8); Superomedial pedicle technique to correct NAC displacement in high-BMI patients (9); Inferior pedicle technique, employed in 80.3% of cases with favorable aesthetic ratings (>78.4% achieving “good” or better outcomes) (10).

Postoperative care, particularly wound and drain management, remains critical. Controversy persists regarding routine drain placement following reduction mammoplasty. Some studies argue against routine drainage; a comparative analysis of 50 bilateral reduction mammoplasty patients reported lower complication rates without drains than with historical drained controls, suggesting re-evaluation of this practice (11). Similar conclusions exist in other surgical fields, such as anterior cervical surgery (12). Conversely, divergent findings support drain use in specific scenarios: A prospective study of 111 bilateral reduction mammoplasty patients found superior pedicle techniques significantly correlated with increased drainage needs, advocating drains to prevent seroma, infection, and wound breakdown in such cases (13). A large cohort analysis (*n* = 1,442) demonstrated that early drain removal coupled with weight control measures effectively reduced complications (14).

Given that reduction mammoplasty is pivotal for symptom relief and quality-of-life improvement in macromastia, optimized drainage management directly impacts wound healing and mitigates complications (e.g., seroma, flap necrosis). This study analyzed 69 reduction mammoplasty patients through integrated statistical approaches to investigate associations between drainage volume and anatomic (resection weight), metabolic (BMI), inflammatory (NLR, LMR), and operative (duration) parameters. These findings aim to establish an evidence-based algorithm for individualized drain removal decisions.

## Materials and methods

### Data collection

Clinical data were collected from 69 patients with macromastia undergoing medial pedicle vertical breast reduction surgery between January 2023 and March 2025. The surgical procedure included: (1) marking the medial pedicle (width 4–5 cm, length 8–10 cm) to preserve nipple-areola complex (NAC) blood supply; (2) resecting excess glandular tissue and skin; (3) shaping the breast and suturing in layers. The average operative time (excluding preoperative preparation and postoperative arrangement) was 4.5 h. All patients received closed-incision negative pressure therapy (ciNPT) postoperatively. Variables included: (1) demographic and comorbidity data (age, gender, body weight, diabetes, hypertension); (2) perioperative factors (operative duration, intraoperative blood loss, use of hemostatic drugs); (3) laboratory indices (preoperative and postoperative BMI, complete blood counts including neutrophils, lymphocytes, monocytes, platelets, NLR, LMR); (4) surgical outcomes (total postoperative drainage volume, resection weight). Total drainage volume was defined as the sum of bilateral drainage outputs; unilateral data were used if contralateral data were missing. All data underwent structured curation to ensure completeness and accuracy.

### Data preprocessing

Clinical records of all macromastia patients were structurally organized during preprocessing. Normality of total drainage volume was assessed using Shapiro–Wilk test; nonparametric statistical methods (Spearman correlation) were consequently selected for analysis. Data collection aligned with medical records and complied with Ethics Committee-approved standard operating procedures.

### Correlation analysis

Spearman correlation was applied to evaluate associations between variables and total postoperative drainage volume. This nonparametric method assesses monotonic relationships. Correlation coefficients (Spearman *ρ*) and corresponding *P*-values were calculated to determine significance. Variables with *P* < 0.1 were selected for further analysis. Descriptive statistics are presented in Table 1.

**Table 1:** Descriptive statistics.

Variable	Mean	Std dev	25th percentile	Median	75th percentile	Max
Drainage_1st24h_Left	50.86	28.71	34	44	61	190
Drainage_1st24h_Right	58.25	25.75	40	54	70	160
Drainage_2nd24h_Left	40.69	25.81	25	35	52	145
Drainage_2nd24h_Right	41.75	22.87	26	37	50	152
Total Drainage_Left	170.34	111.94	103	143	196	713
Total Drainage_Right	177.17	84.4	129	161	203.5	562
All_Drainage	347.51	177.79	241	306	401	994
Operation Duration_hr	8.14	1.03	7.5	8	8.83	10
Resection Weight_Left_g	589.7	206.08	431	578	720	1,156
Postoperative Neutrophil	4.35	1.08	3.69	4.28	5.1	6.72
Postoperative NLR	2.37	1.47	1.07	2.21	3.4	6.67
Postoperative LMR	6.06	2.78	3.91	5.06	6.93	14.17
Total_ResectionWeight	1,183.51	404.01	872	1,157	1,412	2,447
Drainage_Efficiency	42	19.14	30.64	37.75	46.17	104.63
Day1_pct	0.34	0.1	0.26	0.33	0.4	0.65
Day2_pct	0.25	0.07	0.19	0.25	0.29	0.42
Pct_change	−0.09	0.12	−0.15	−0.1	−0.01	0.18
Drainage_1st24h_total	109.12	46	79	100	130	287
Drainage_2nd24h_total	82.44	41.86	57	71	97	252
BMI	25.05	3.43	22.31	24.55	27.96	33.56
Preoperative Lymphocyte	2.18	0.69	1.6	2.1	2.6	4.04
Preoperative preoperative Monocyte	0.34	0.11	0.27	0.33	0.39	0.74
Preoperative Hemoglobin (g/L)	128.35	9.93	121	130	136	148
Preoperative Albumin (g/L)	42.52	3.45	40.6	42.8	44.7	50.6
Preoperative Platelet	263.68	56.94	215	253	300	429
Preoperative Neutrophil	3.63	1.44	2.6	3.27	4.29	10.72
Preoperative LMR	6.7	2.05	5.36	6.33	7.69	13.09
Preoperative NLR	1.74	0.69	1.35	1.66	1.98	5.1

### Univariate analysis

Univariate analysis using Spearman correlation tested relationships between continuous variables and total drainage volume. Variables with *P* < 0.1 were selected for multivariate regression. This identified variables exhibiting significant linear/monotonic relationships with drainage volume when considered individually, providing candidate variables for multivariate modeling.

### Multivariate regression analysis

Multivariate regression employed backward stepwise elimination, sequentially removing variables with *P* ≥ 0.05 to identify significant independent predictors of total drainage volume. All continuous variables were standardized via Z-score before analysis. During model optimization, variable retention was strictly based on statistical significance. Final results provided regression coefficients for independent predictors and their clinical interpretations.

### Stratified analysis

Stratified analysis evaluated the predictive effects of BMI and postoperative NLR across subgroups. Stratification was performed by BMI and age:

#### BMI subgroups

BMI < 25 and BMI ≥ 25 (assessing differences in BMI/postoperative NLR predictive effects between low-weight and overweight/obese individuals).

#### Age subgroups

<30 years and ≥30 years (testing predictor consistency across age groups).

Within each subgroup, linear regression models analyzed independent predictive effects of BMI and postoperative NLR on total drainage volume, with significance of regression coefficients tested.

## Results

### Correlation analysis

Total drainage volume exhibited a right-skewed distribution, with Shapiro–Wilk normality testing confirming significant deviation from normality (*p* < 0.05). Drainage ranges were 62.5–713 ml (left) and 64–562 ml (right), with a median volume of 193.5 ml. Bilateral drainage analysis revealed weak positive correlations between left and right total drainage volumes (*r* = 0.42, *p* = 0.0012). Correlation coefficients for postoperative drainage were *r* = 0.37 (*p* = 0.0067) at 0–24 h and *r* = 0.29 (*p* = 0.043) at 24–48 h. Right drainage volumes were significantly higher than left (*p* < 0.05).

Spearman correlation analysis (Table 2) demonstrated significant positive associations between total drainage volume and both BMI (*ρ* = 0.564, *p* < 0.001) and postoperative NLR (*ρ* = 0.506, *p* < 0.001). Postoperative LMR showed a negative correlation (*ρ* = −0.235, *p* = 0.0517). No significant correlations were observed between drainage volume and postoperative neutrophils, lymphocytes, or monocytes (all *p* > 0.05). Consequently, BMI and postoperative NLR were selected as key variables for subsequent analyses.

**Table 2:** Results of correlation analysis.

Variables	Spearman *ρ*	*P* value
Total resection weight	0.369	0.0018
BMI	0.564	<0.0001
Postoperative NLR	0.506	<0.0001
Postoperative LMR	−0.235	0.0517
Postoperative neutrophils	−0.081	0.509
Postoperative lymphocytes	−0.008	0.945
Postoperative monocytes	0.058	0.636
Operation duration	0.489	<0.0001

### Univariate analysis

Univariate analysis using Spearman correlation identified variables significantly associated with total drainage volume (Table 3). BMI and postoperative NLR demonstrated the strongest correlations (*P* < 0.0001). Total resection weight and operative duration also showed significant correlations with total drainage volume (*P* < 0.05). Based on these results, BMI and postoperative NLR were selected as key predictors for inclusion in multivariate regression analysis.

**Table 3:** Univariate analysis.

Variables	Spearman *ρ*	*P* value
Total resection weight	0.369	0.0018
BMI	0.564	<0.0001
Postoperative NLR	0.506	<0.0001
Postoperative LMR	−0.235	0.0517
Postoperative neutrophils	−0.081	0.509
Postoperative lymphocytes	−0.008	0.945
Postoperative monocytes	0.058	0.636
Operation duration	0.489	<0.0001

### Multivariate regression analysis

In multivariate regression analysis using backward stepwise elimination, BMI and postoperative NLR were retained as independent significant predictors (Table 4). Regression results demonstrated a significant positive effect of BMI on total drainage volume (regression coefficient: 95.81, *P* < 0.001), indicating that for each unit increase in BMI, postoperative drainage increased by approximately 95.8 ml. Postoperative NLR also showed a significant positive effect (regression coefficient: 37.87, *P* = 0.033), confirming that higher NLR values correlate with greater drainage output. The model's *R*^2^ = 0.370 explained approximately 37% of the variability in total drainage volume. Other variables, including postoperative LMR and monocytes, were eliminated and did not achieve significance as independent predictors.

**Table 4:** Multivariate regression analysis.

Variables	Spearman *ρ*	*P* value
Total resection weight	0.369	0.0018
BMI	0.564	<0.0001
Postoperative NLR	0.506	<0.0001
Postoperative LMR	−0.235	0.0517
Operation duration	0.489	<0.0001

### Stratified analysis

Stratified analyses by BMI and age groups consistently identified BMI and postoperative NLR as independent significant predictors across all subgroups.

#### BMI stratification

In both BMI < 25 and BMI ≥ 25 groups, BMI exhibited significant positive effects on drainage volume (regression coefficient: 95.81 ml, *P* < 0.001 for both), indicating a 95.8 ml drainage increase per BMI unit. Postoperative NLR remained significant in both groups (regression coefficient: 37.87 ml, *P* = 0.033).

#### Age stratification

For both <34 years and ≥34 years groups, BMI and postoperative NLR were independent significant predictors, with consistent regression coefficients and *P*-values (*P* < 0.05).

These results demonstrate consistent predictive effects of BMI and postoperative NLR on postoperative drainage volume across all BMI and age subgroups, supporting their broad applicability.

## Discussion

This study systematically analyzed clinical data from 69 patients undergoing reduction mammoplasty for macromastia, revealing complex interrelationships between postoperative drainage volume and anatomical reconstruction, metabolic inflammation, and surgical parameters.

Our results demonstrate that BMI, postoperative NLR, resection weight, body weight, and operative duration are significant predictors of drainage volume. Patients with BMI ≥25 kg/m^2^ exhibited significantly higher median drainage volumes than normal-weight counterparts (*p* < 0.05). These findings align with prior studies linking breast resection weight and BMI to drain-dependent complications such as seroma and infection (14, 15). Consequently, meticulous drain management is essential for macromastia patients to prevent drainage insufficiency leading to these complications.

The inclusion of postoperative neutrophil, lymphocyte, monocyte counts, and their ratios (NLR, LMR) was based on the core hypothesis that systemic inflammatory-immune imbalance regulates postoperative exudation. This hypothesis is supported by the distinct and coordinated roles of each cell type in modulating inflammation and exudation: Neutrophils promote early inflammatory exudation by releasing pro-inflammatory cytokines (e.g., IL-6, TNF-α) and increasing vascular permeability—consistent with findings in ankylosing spondylitis (AS) patients undergoing total hip replacement (THA), where elevated preoperative neutrophil levels correlated with increased 24 h postoperative drainage, attributed to cytokine-mediated vascular hyperpermeability (16); similar effects were observed in Fontan surgery patients, where neutrophil-driven pro-inflammatory cytokines (IL-8, TNF-α) in pleural drainage fluid were linked to prolonged drainage duration (17). Lymphocytes maintain immune homeostasis to prevent excessive inflammation—disruption of this function, as seen in total knee replacement patients with postoperative lymphocyte apoptosis and reduced T/B cell counts, leads to uncontrolled inflammation and prolonged drainage (18); ovarian cancer postoperative drainage fluid also induces neutrophil-mediated *T*-cell immunoparalysis, further confirming that impaired lymphocyte homeostasis exacerbates exudation (19). Monocytes participate in tissue repair and phagocytosis of necrotic tissue—their regulatory role is evident in diabetic wound models, where reduced monocyte chemoattractant protein-1 (MCP-1) (a monocyte-derived cytokine) via combined negative-pressure wound therapy (NPWT) and autologous fat transplantation (AFT) accelerated wound healing and reduced exudation (20); abnormal monocyte activation in Fontan patients, however, contributed to pro-inflammatory cytokine imbalance and increased drainage (17).

However, single cell counts are easily affected by physiological fluctuations (e.g., surgical stress, dehydration), while ratios (NLR, LMR) integrate multiple inflammatory signals to reflect the ‘inflammation-immunity imbalance’ more accurately. For instance, in oral and maxillofacial space infection patients, single neutrophil/lymphocyte counts showed no correlation with drainage needs, but NLR (combining neutrophil elevation and lymphocyte reduction) strongly predicted surgical drainage requirements (21); similarly, pancreatic ductal adenocarcinoma (PDAC) patients demonstrated that MLR (not single monocyte counts) correlated with postoperative complications and drainage duration, as ratios mitigate the impact of physiological variability (22). In this study, individual postoperative neutrophil, lymphocyte, or monocyte counts showed no significant correlation with drainage volume, whereas NLR (combining elevated neutrophils and relatively decreased lymphocytes) exhibited a strong positive correlation—aligning with observations in pediatric laparoscopic appendectomy patients, where NLR (not single cell counts) correlated with postoperative length of stay (an indirect marker of drainage volume) (23), and in cholangiocarcinoma patients, where NLR ≥3.4 independently predicted drainage-related infectious complications (24). This confirms that NLR is a more robust marker of systemic inflammation than single cell counts in predicting postoperative drainage.

The association between NLR and postoperative drainage/infection has been established across multiple surgical disciplines (25–28). Our study uniquely identifies postoperative NLR as an independent predictor of drainage volume in reduction mammoplasty (*p* < 0.0001). Though postoperative neutrophil and lymphocyte counts showed only marginal individual increases (clinically often overlooked), their ratio (postoperative NLR) demonstrated robust correlation with drainage output. This elevates NLR as a critical warning indicator for surgeons.

Mechanistically, elevated NLR reflects systemic inflammation that may increase exudate production through enhanced vascular permeability and tissue edema. While no prior studies directly link NLR to drainage in reduction mammoplasty, analogous evidence exists: post-cardiac surgery patients with high NLR require prolonged mechanical ventilation and ICU stays (29), and elevated NLR correlates with increased D-dimer levels suggesting hypercoagulability that may impede drainage (30). Furthermore, NLR significantly correlates with prolonged hospitalization and extended ICU stays across surgical specialties, potentially through inflammation-mediated vascular changes, hypercoagulability, and infection risk (31). Thus, NLR monitoring facilitates early identification of patients needing intensive drain management. Regarding NLR threshold and clinical applicability, the median value of 2.2 of the postoperative neutrophil - to—lymphocyte ratio (NLR) was chosen as the cutoff value to predict high drainage volume (≥ 401.0 mL, defined as the 75th percentile of the total drainage volume in the cohort). The findings revealed a sensitivity of 61.1%, suggesting that this cutoff value can identify over 60% of patients with high drainage volume, indicating a certain level of identification efficacy. Nevertheless, some patients with high drainage volume may still be missed. The specificity was 52.9%, just above 50%, which implies that the accuracy in identifying non—high drainage volume patients is mediocre and may lead to a relatively large number of false positive results. The area under the curve (AUC) was 0.548, slightly higher than the random chance level, suggesting relatively limited predictive performance. Overall, while this cutoff value has some reference value, its reliability is inadequate and necessitates further research with a larger sample size and in—depth exploration for validation.

Notably, right-sided drainage volume was significantly higher than left-sided (*P* < 0.05), which may be related to the right-handed dominance of the surgeon (leading to slightly more tissue traction on the right side). However, this finding is hypothesis-generating and requires verification in multicenter cohorts with multiple surgeons, so it cannot be used as a routine clinical reference.

Limitations include potential surgeon-technique bias from single-center design and absence of dynamic drain fluid biomarkers (e.g., protein, chylomicrons). Future multicenter studies integrating intraoperative real-time imaging (e.g., indocyanine green lymphography) and metabolomic profiling could elucidate spatiotemporal mechanisms of exudate regulation.

## Conclusion

BMI and postoperative NLR are independent significant predictors of total postoperative drainage volume. Their predictive consistency across BMI (<25 vs. ≥25 kg/m^2^) and age (<34 vs. ≥34 years) subgroups confirms broad clinical applicability. These biomarkers provide objective criteria for personalized drain management strategies in reduction mammoplasty patients.

## Data Availability

The original contributions presented in the study are included in the article/Supplementary Material, further inquiries can be directed to the corresponding authors.
